# The tight junction protein claudin 6 is a potential target for patient-individualized treatment in esophageal and gastric adenocarcinoma and is associated with poor prognosis

**DOI:** 10.1186/s12967-023-04433-8

**Published:** 2023-08-17

**Authors:** Adrian Georg Simon, Su Ir Lyu, Mark Laible, Stefan Wöll, Özlem Türeci, Uğur Şahin, Hakan Alakus, Luca Fahrig, Thomas Zander, Reinhard Buettner, Christiane Josephine Bruns, Wolfgang Schroeder, Florian Gebauer, Alexander Quaas

**Affiliations:** 1 Institute of Pathology, University Hospital Cologne, Medical Faculty, University of Cologne, Kerpener Str. 62, 50937 Cologne, Germany; 2 BioNTech SE, Mainz, Germany; 3 Department of General, Visceral and Cancer Surgery, University Hospital Cologne, Medical Faculty, University of Cologne, Cologne, Germany; 4 Department of Internal Medicine I, University Hospital Cologne, Medical Faculty, University of Cologne, Cologne, Germany

**Keywords:** Adenocarcinoma, CAR T-cell therapy, Immunotherapy, Claudin, CLDN6, Tight junctions, Esophageal cancer, Gastric cancer

## Abstract

**Background:**

The prognosis of esophageal adenocarcinoma (EAC) and gastric adenocarcinoma (GAC) remains poor, and new therapeutic approaches are urgently needed. Claudin 6 (CLDN6) is an oncofetal antigen that is largely absent in healthy tissues and upregulated in several cancers, making it a promising therapeutical target. In this study, the expression of CLDN6 was assessed in an large Caucasian EAC and GAC cohort.

**Methods:**

RNA-Seq data from 89 EACs and 371 GACs were obtained from The Cancer Genome Atlas project and EAC/GAC cases were stratified by CLDN6 mRNA expression based on a survival-associated cutoff. For groups with CLDN6 expression above or below this cutoff, differential gene expression analyses were performed using DESeq, and dysregulated biological pathways were identified using the Enrichr tool. Additionally, CLDN6 protein expression was assessed in more than 800 EACs and almost 600 GACs using a CLDN6-specific immunohistochemical antibody (clone 58-4B-2) that is currently used in Phase I/II trials to identify patients with CLDN6-positive tumors (NCT05262530; NCT04503278). The expression of CLDN6 was also correlated with histopathological parameters and overall survival (OS).

**Results:**

EACs and GACs with high CLDN6 mRNA levels displayed an overexpression of pathways regulating the cell cycle, DNA replication, and receptor / extracellular matrix interactions. CLDN6 protein expression was associated with shorter OS in EAC and GAC, both in treatment-naïve subgroups and cohorts receiving neoadjuvant therapy. In multivariate analysis, CLDN6 protein expression was an independent adverse prognostic factor in EAC associated with a shorter OS (HR: 1.75; p = 0.01) and GAC (HR: 2.74; p = 0.028).

**Conclusions:**

High expression of CLDN6 mRNA is associated with the dysregulation of distinct biological pathways regulating cell growth, proliferation, and cell–matrix interactions. Clinically, the expression of CLDN6 protein is a valuable adverse prognostic marker in EAC and GAC.

## Background

Esophageal adenocarcinoma (EAC) and gastric adenocarcinoma (GAC) are the two most common cancers of the upper gastrointestinal tract. Together they accounted for approximately 13% of all worldwide cancer deaths in 2020 [1]. Despite recent advances in surgery and perioperative systemic treatment approaches, the prognosis of EAC, especially if inoperable or advanced, remains dismal. Patients treated with neoadjuvant (radio-) chemotherapy and esophagectomy have a 5-year survival rate of 43%. When surgery is not an option and the disease is surgically irresectable, survival is usually only a few months [2–4]. In GAC, the prognosis depends on localization and infiltration of the tumor as well as the presence of distant metastasis. While well-differentiated tumors limited to the mucosa and submucosa can be cured by mucosectomy with a 5-year survival of up to 97% [5], advanced GAC has a very poor prognosis. Even if treated with gastrectomy and perioperative chemotherapy, patients with advanced gastric cancer have a 5-year survival of only 36% [6]. This demonstrates an urgent need for new treatment options in advanced EAC and GAC.

Claudin proteins are components of tight junctions and have been identified as potential therapeutic targets in several cancers. Their dysregulation and role in tumorigenesis, invasion, and metastasis have been investigated over the last decade [7], leading to promising experimental and clinical approaches. One of the members of this family, claudin 6 (CLDN6), is expressed only in the fetal stage [8], forms primitive tight junctions and is completely silenced after completion of fetal organogenesis. CLDN6 expression is tightly suppressed in healthy adult tissues, but aberrantly activated in various solid tumor types [9–15]. High and frequent CLDN6 expression is found in germ cell tumors, epithelial ovarian cancer, endometrial carcinoma, and various other cancer types, including rare malignancies. CLDN6 is involved in intracellular signaling and consists of four transmembrane domains that can bind to other signaling proteins as well as cytoskeletal components on the intracellular side [16, 17].

In GAC, first studies with smaller cohorts demonstrated that the expression of CLDN6 is associated with tumor cell proliferation and increased invasiveness; additionally, a correlation between CLDN6 expression and reduced overall survival was observed [18, 19]. The biological function in tumorigenesis and prognostic impact of CLDN6 in EAC, however, are unknown. In this study, we analyzed the biological profile of EAC and GAC cases with high levels of CLDN6 mRNA expression. We performed differential gene expression analysis using openly available data from The Cancer Genome Atlas (TCGA) project followed by pathway enrichment analyses. Additionally, the prognostic impact of CLDN6 protein expression was assessed in a large European EAC and GAC patient cohort.

## Materials and methods

### TCGA cohort of EACs and GACs

RNA-Seq data acquisition, analysis and visualization were performed with R (v.4.2.2) and RStudio (v. 2022.12.0 + 353). The Broad Institute Firehose GDC portal was used for retrieval of raw counts, RSEM-normalized transcript per million (TPM) counts, and clinical data (https://gdac.broadinstitute.org/) [20]. These openly available patient cohorts consisted of 89 cases of esophageal adenocarcinoma (EAC), 371 cases of gastric adenocarcinoma (GAC), ten cases of benign esophageal tissue, and 34 samples of gastric benign tissue for comparison. The histopathological data from the acquired EAC and GAC cases are displayed in Table 1.

**Table 1:** TCGA cohorts of EAC and GAC

		EAC (n = 89)	GAC (n = 371)
		n (%)	n (%)
Sex			
	Male	77 (86.5)	242 (65.2)
	Female	12 (13.5)	129 (34.8)
Median age (range)		69 years (28–86 years)	67 years (30–90 years)
pT			
	pTx	15 (16.8)	4 (1.1)
	pT0	1 (1.1)	0 (0.0)
	pT1	23 (25.8)	16 (4.3)
	pT2	11 (12.3)	80 (21.6)
	pT3	38 (42.7)	174 (46.9)
	pT4	1 (1.1)	97 (26.1)
pN			
	pNx	16 (18.0)	11 (3.0)
	pN0	22 (24.7)	115 (31.0)
	pN1 +	51 (57.3)	245 (66.0)
M			
	Mx	32 (36.0)	5 (1.3)
	M0	52 (58.4)	347 (93.5)
	M1	5 (5.6)	19 (5.1)
TCGA molecular subtype^a^			
	NA	–	131 (35.3)
	EBV	–	24 (6.5)
	MSI-high	–	48 (12.9)
	GS	–	48 (12.9)
	CIN	–	120 (32.3)
CLDN6 expression^b^			
	> Best cutoff	14 (15.7)	68 (18.3)
	< Best cutoff	75 (84.3)	303 (81.7)
Her2/ERBB status			
	Amplified	47 (5.9)	
	Not amplified	518 (64.5)	
	NA	238 (29.6)	

*CIN* chromosome instability-associated GAC, *CLDN6* claudin 6, *EAC* esophageal adenocarcinoma, *EBV* EBV-associated GAC, *GAC* gastric adenocarcinoma, *GS* Genomic stable GAC, *M* distant metastasis, *median age* median age at diagnosis, *pT* tumor stage, *pN* lymph node stage, *MSI-high* microsatellite-instable GAC, *NA* not available, *TCGA* The Cancer Genome Atlas

^a^Molecular subtype according to the molecular TCGA subtype classification of GAC [29]

^b^CLDN6 expression (TPM, RSEM-normalized) dichotomized by the best cutoff value for a significant difference in overall survival in a log-rank test (see methods)

### RNA-Seq data analysis

Tumor cohorts were dichotomized by the RSEM-normalized median expression of CLDN6 (TPM) as well as the best survival-associated cutoff value for RSEM-normalized expression, which was identified using the openly available Cutoff Finder tool: This cutoff value of CLDN6 mRNA expression allows the most significant stratification of the case cohort by overall survival, using a log rank test with a p-value < 0.05 considered significant [21, 22]. Tumor cohorts of EAC and GAC, dichotomized by this survival-associated cutoff value, were then further analyzed with the Bioconductor software package DESeq2 (v.3.16). Genes that were significantly dysregulated compared to benign tissue were identified using the Wald test with Bonferroni correction for multiple testing [23]. For noise reduction and further normalization, a log fold change shrinkage method (apeglm package v.3.16) was used [24]. An adjusted p-value of < 0.05 was considered significant.

### Enrichr pathway analysis in EAC and GAC specimens

The EnhancedVolcano package was used to generate volcano plots [25]. Significantly upregulated genes were identified with a log2 fold change (log2FC) of ≥ 1.5, and downregulated genes were identified with a log2FC of ≤ -1.5. These gene lists were then called in the Enrichr database for subsequent gene set and pathway analysis to identify differences between tumors with low and high CLDN6 expression [26].

### Patient cohort of EAC and GAC for immunohistochemistry

For immunohistochemical analysis of CLDN6 expression, patient tissue from 803 cases of EAC and 589 cases of GAC were obtained for which surgically removed tumor specimens, histopathological data and follow-up data were available (Table 2). All patients were treated between 1996 and 2020 at the Department of General, Visceral and Cancer Surgery at the University Hospital Cologne, Germany. For EAC, patients were surgically treated with right transthoracic esophagectomy and a two-field lymphadenectomy (mediastinal and abdominal lymph nodes). The intestinal passage was reconstructed with a high intrathoracic esophagogastrostomy as described previously [27]. Patients with GAC were treated with a subtotal distal or total gastrectomy with trans-hiatal resection of the distal esophagus in case of a Siewert II-tumor, followed by lymphadenectomy (level D2). A Roux-en-Y jejunal loop with gastrojejunostomy was the method for intestinal reconstruction. In EAC, neoadjuvant therapy consisted of either radiochemotherapy according to the Chemo-radiotherapy for Oesophageal Cancer followed by Surgery Study (CROSS) protocol (carboplatin, paclitaxel and intensity-modulated radiotherapy) or perioperative chemotherapy (Fluorouracil, Leucovorin, Oxaliplatin and Docetaxel: FLOT). For the treatment of patients with GAC three different perioperative regimens were used in the last two decades: Cisplatin, 5-Fluorouracil and Leucovorin (PFL), Medical Research Council Adjuvant Gastric Infusional Chemotherapy (Epirubicin, Cisplatin, Fluorouracil: MAGIC) and FLOT. Most patients were treated with MAGIC and FLOT protocols according to national German guidelines.

**Table 2:** Cohorts of EAC and GAC for immunohistochemistry analysis of CLDN6

		EAC (n = 803)	GAC (n = 589)
		n (%)	n (%)
Sex			
	Male	706 (87.9)	397 (67.4)
	Female	97 (12.1)	192 (32.6)
Median age (range)		64 years (28–92 years)	67 years (18–91 years)
pT			
	pT1	141 (17.6)	81 (13.8)
	pT2	144 (17.9)	178 (30.2)
	pT3	488 (60.8)	236 (40.1)
	pT4	30 (3.7)	94 (15.9)
pN			
	pNx	4 (0.5)	1 (0.1)
	pN0	323 (40.2)	201 (34.1)
	pN1 +	476 (59.3)	387 (66.8)
L			
	Lx	137 (17.1)	75 (12.7)
	L0	350 (43.6)	192 (32.6)
	L1	316 (39.3)	322 (54.7)
V			
	Vx	132 (16.4)	75 (12.7)
	V0	586 (73.0)	434 (73.7)
	V1	85 (10.6)	80 (13.6)
Pn			
	Pnx	131 (16.0)	75 (12.7)
	Pn0	508 (63.3)	386 (65.5)
	Pn1	166 (20.7)	128 (21.7)
M			
	Mx/M0	760 (94.65)	475 (81.6)
	M1	43 (2.8)	114 (19.4)
Neoadjuvant treatment^a^			
	Yes	514 (36.0)	195 (33.1)
	No	289 (64.0)	394 (66.9)
AJCC Grade^b^			
	NA	6 (2.1)	2 (0.5)
	1	2 (0.7)	3 (0.7)
	2	143 (49.5)	130 (33.0)
	3	133 (47.7)	259 (65.8)
Her2 status (ERBB)			
	Amplified	47 (5.9)	54 (9.2)
	Not amplified	518 (64.5)	409 (69.4)
	NA	238 (29.6)	126 (21.4)
TCGA molecular subtype^c^			
	NA	–	115 (19.5)
	EBV	–	24 (4.1
	MSI-high	–	42 (7.1)
	GS	–	50 (8.5)
	CIN	–	358 (60.8)

*AJCC* American Joint Committee on Cancer, *CIN* Chromosome instability-associated GAC, *CROSS* Chemo-rradiotherapy for Oesophageal cancer followed by Surgery Study, *EAC* esophageal adenocarcinoma, *EBV* EBV-associated GAC, *FLOT* fluorouracil-leucovorin-oxaliplatin-docetaxel, *GAC* Gastric adenocarcinoma, *GS* Genomic stable GAC, *L* Lymph vessel invasion, *M* distant metastasis, *median age* median age at diagnosis, *MSI-high* microsatellite-instable GAC, *NA* not available, *pT* tumor stage, *pN* lymph node stage, *Pn* perineural invasion, *TCGA* The Cancer Genome Atlas, *V* blood vessel invasion

^a^Neoadjuvant therapy usually consisted of CROSS or FLOT regimen

^b^AJCC Grading was only performed for tumors without neoadjuvant therapy

^c^Molecular subtype according to the molecular TCGA subtype classification of GAC [29]

In the first two years after surgery, a clinical follow-up was performed every three months, followed by annual check-ups. The clinical follow-up included a detailed check of the patient’s medical history, physical examination, an ultrasound scan of the abdomen, a chest X-ray, and if required, additional diagnostics.

Written consent was obtained by all patients for the scientific usage of tissue specimens. The ethics committee of the University Hospital of Cologne approved the project (ethics committee number: 21-1146).

### Tissue microarray generation and immunohistochemical staining

Tissue microarrays were generated using formalin-fixed, paraffin-embedded, surgically resected tumor specimens as reported previously [28]. Shortly summarized, one tissue core with a diameter of 1.2 mm was transferred to the recipient paraffin block with a self-constructed, semi-automated precision instrument. Placenta tissue was included as a control.

To assess CLDN6 expression in EAC and GAC samples, manual immunohistochemical staining was performed according to the manufacturer’s instructions using the CE-IVD kit CLAUDENTIFY^®^ 6 (BioNTech Diagnostics GmbH, Mainz, Germany), which uses the CLDN6-specific antibody clone, 58-4B-2.

### Assessment of CLDN6 expression in EAC and GAC

The extent of CLDN6 expression was determined using a semi-quantitative approach. Only membranous staining (complete and incomplete) was considered. The staining intensity as well as the percentage of stained cells were assessed (0 = negative, 1 +  = weak, 2 +  = moderate, 3 +  = strong). Tumors were classified as CLDN6 negative if the tumor cells displayed weak staining (1 + , only visible at higher magnification) or if < 5% of the tumor cells displayed CLDN6 staining with an intensity of ≥ 2 + . If an intensity of ≥ 2 + was seen in ≥ 5% but < 50% of tumor cells, the sample was classified as low positive. If CLDN6 was expressed with an intensity ≥ 2 + in ≥ 50% tumor cells, the sample was classified as high positive.

For GAC and EAC, the Her2 status (ERBB) and, in case of GAC, the molecular subtype according to the TCGA classification was identified using routine diagnostic protocols of the molecular pathology laboratory at Cologne University Hospital as described previously, including immunohistochemistry and Epstein-Barr encoding region in situ hybridization [28].

### Statistical analysis and survival analysis

All data processing and statistical analysis, including survival analysis and visualization, were performed with R (v.4.2.2) and Rstudio (v. 2022.12.0 + 353) with common free packages including survival (v.3.4-0), survminer (v.0.4.9), and ggplot2 (v.3.4.0).

Interdependencies between clinical data, histopathological data and CLDN6 expression were evaluated using Fisher’s exact test and Spearman’s correlation test.

To assess the correlation between CLDN6 expression in EAC and GAC and its association with the outcome, overall survival (OS) was evaluated from the date of surgery until death (of any cause). Kaplan Meier curves were generated and a log-rank test was used. Patient data with no events or patients lost to clinical follow-up were censored at the last known date. For multivariate analysis, covariates were implemented using the ENTER method in a Cox proportional hazard model. Covariates which were significant in univariate analysis were included in the multivariate model. A p-value < 0.05 considered to be significant in all tests.

## Results

### Higher CLDN6 mRNA expression is associated with significantly shorter OS in the TCGA cohorts

**Fig. 1:**
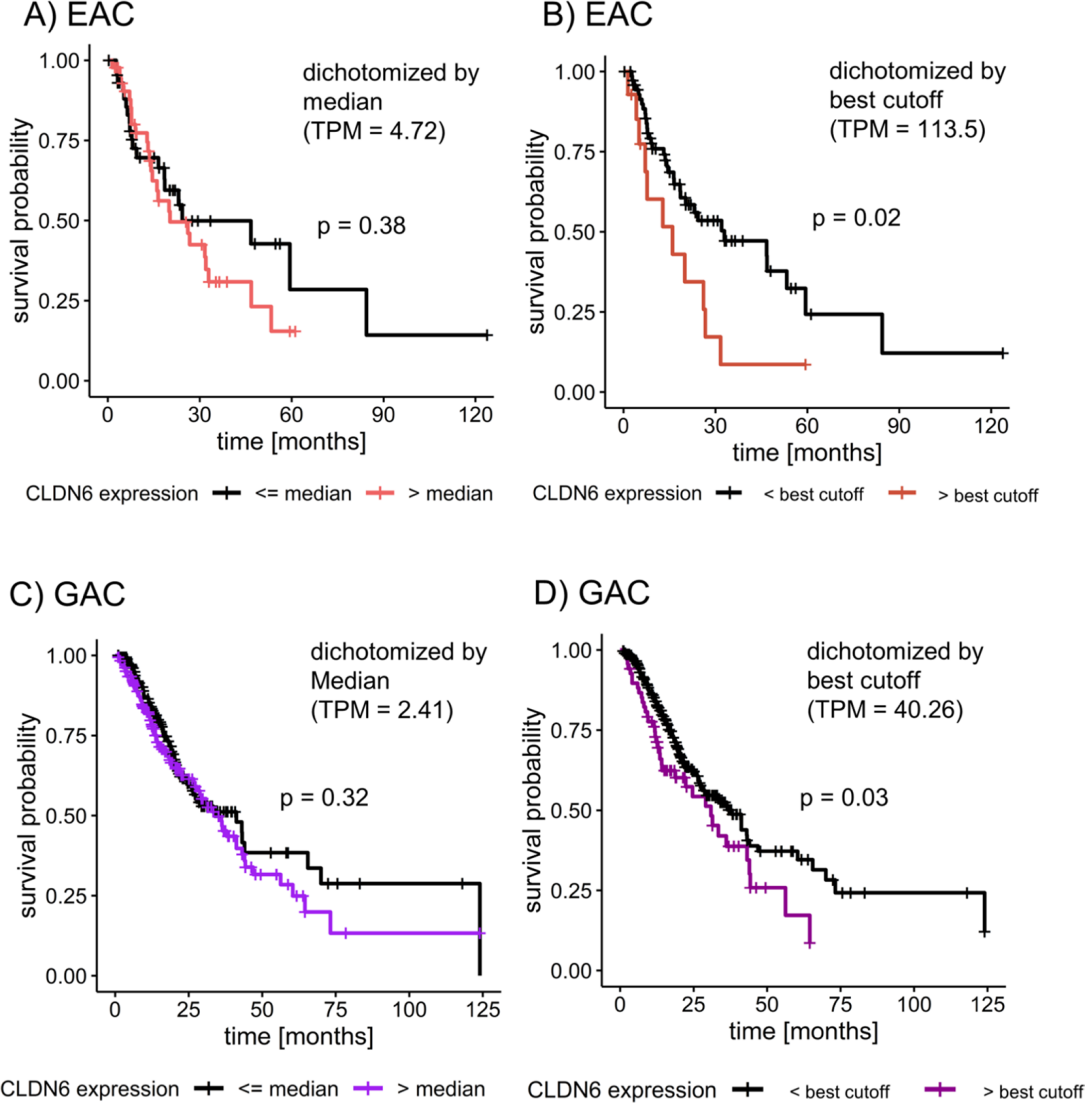
Higher CLDN6 mRNA expression is associated with shorter OS and a poor prognosis. **A** OS for 89 EAC specimens dichotomized by the median CLDN6 expression (TPM, RSEM-normalized). **B** OS in EACs dichotomized by the best survival-associated cutoff identified by the Cutoff Finder tool; n > best cutoff = 14, n < best cutoff = 75. **C** OS in GAC specimens (n = 371) stratified by the median CLDN6 expression; **D** OS differences in GAC patients, dichotomized by the best survival-associated cutoff identified by the Cutoff Finder tool; n > best cutoff = 68, n < best cutoff = 303. CLDN6 = claudin 6, *EAC* esophageal adenocarcinoma, *GAC* gastric adenocarcinoma, *OS* overall survival, *TCGA* The Cancer Genome Atlas, *TPM* transcripts per million. A p-value < 0.05 (log-rank test) was considered significant

EAC and GAC TCGA cohorts were stratified based on high or low CLDN6 mRNA expression (TPM, RSEM-normalized) to compare overall survival (OS) between both groups. When dichotomizing EAC and GAC cohorts according to the median CLDN6 expression, no significant difference in OS was detected (Fig. 1A, C). Overall, the expression of CLDN6 in EAC and GAC was variable (EAC: median TPM = 4.72; range 0-3361.91; GAC: median TPM = 2.41, range 0–19598).

For EAC, the Cutoff Finder tool identified the best overall survival-associated cutoff for CLDN6 mRNA expression (TPM = 113.5). When subdivided by this value, patients with CLDN6 expression above the cutoff (n = 14) had a significantly shorter OS than patients with CLDN6 expression below the cut-off (n = 75) (16.0 months vs. 32.9 months; log-rank test p = 0.02; Fig. 1B).

We did not observe any correlation between dichotomized CLDN6 expression and tumor stage (pT), lymph node stage (pN), metastasis stage (pM), age, and sex (data not shown). High levels of CLDN6 were an adverse prognostic factor in the univariate analysis in the TCGA cohort of EAC (HR: 2.21; 95% CI 1.12–4.39; p = 0.02), but not in the multivariate analysis (Table 3).

**Table 3:** Multivariate analysis for CLDN6 mRNA expression in EAC and GAC

	EAC		GAC	
Covariate	HR (95% CI)	p-value	HR (95% CI)	p-value
Age	1.0 (0.96–1.03)	0.81	1.02 (1.0–1.04)	**0.01**
pT	1.17 (0.65–2.02)	0.60	1.24 (0.97–1.57)	0.08
pN	3.91 (1.32–11.64)	**0.01**	1.53 (1.01–2.34)	**0.047**
M	4.91 (1.55–15.61)	**0.007**	1.82 (1.0–3.32)	0.053
CLDN6 > best cutoff	0.63 (0.19–2.02)	0.44	1.50 (1.01–2.21)	**0.042**

The bold values represent significant p-values (< 0.05)

*CI* confidence interval, *CLDN6* claudin 6, *EAC* esophageal adenocarcinoma, *GAC* gastric adenocarcinoma, *HR* hazard ratio, *pN* lymph node stage, *M* metastasis stage, *pT* Tumor stage

In the GAC TCGA cohort, patients with CLDN6 expression over the best cut-off (TPM = 40.26;

n = 68) had significantly shorter OS than patients with CLDN6 expression below the best cut-off (n = 303) (30.9 months vs. 37.9 months; p = 0.03; Fig. 1D). High levels of CLDN6 expression were associated with an adverse outcome in the univariate analysis (HR: 1.51; 95% CI 1.04–2.29; p = 0.03) and in the multivariate analysis (Table 3).

### GAC cases with high CLDN6 mRNA expression from the TCGA database commonly belong to the chromosome-instable (CIN) subgroup

For 240/371 GAC cases (64.7%), molecular subtype classification information was available in the TCGA database [29]. Of these 240 cases, 24/240 were Epstein-Barr Virus (EBV)-associated tumors (10%), 48/240 tumors were of the genome stable subtype (20%), 48/240 tumors had high levels of microsatellite instability (MSI-high-subtype; 20%), and 120/240 tumors belonged to the CIN subtype (50%). For the 40 tumors above the best cut-off for CLDN6 expression, 35 belonged to the CIN subgroup (87.5%), four to the genome stable subgroup (10%), and one tumor belonged to the EBV-associated group (2.5%). For the 200 tumors below the best cut-off point, 85 were in the CIN subgroup (42.5%), 23 were EBV-associated (11.5%), 44 were genome stable (22%), and 48 were of the MSI-high subtype (24%). Tumors with higher CLDN6 expression and shorter OS therefore belonged mostly to the CIN type (p < 0.001, Fisher’s exact test).

### EAC and GAC cases from the TCGA database with high and low CLDN6 mRNA expression display distinct biological profiles

**Fig. 2:**
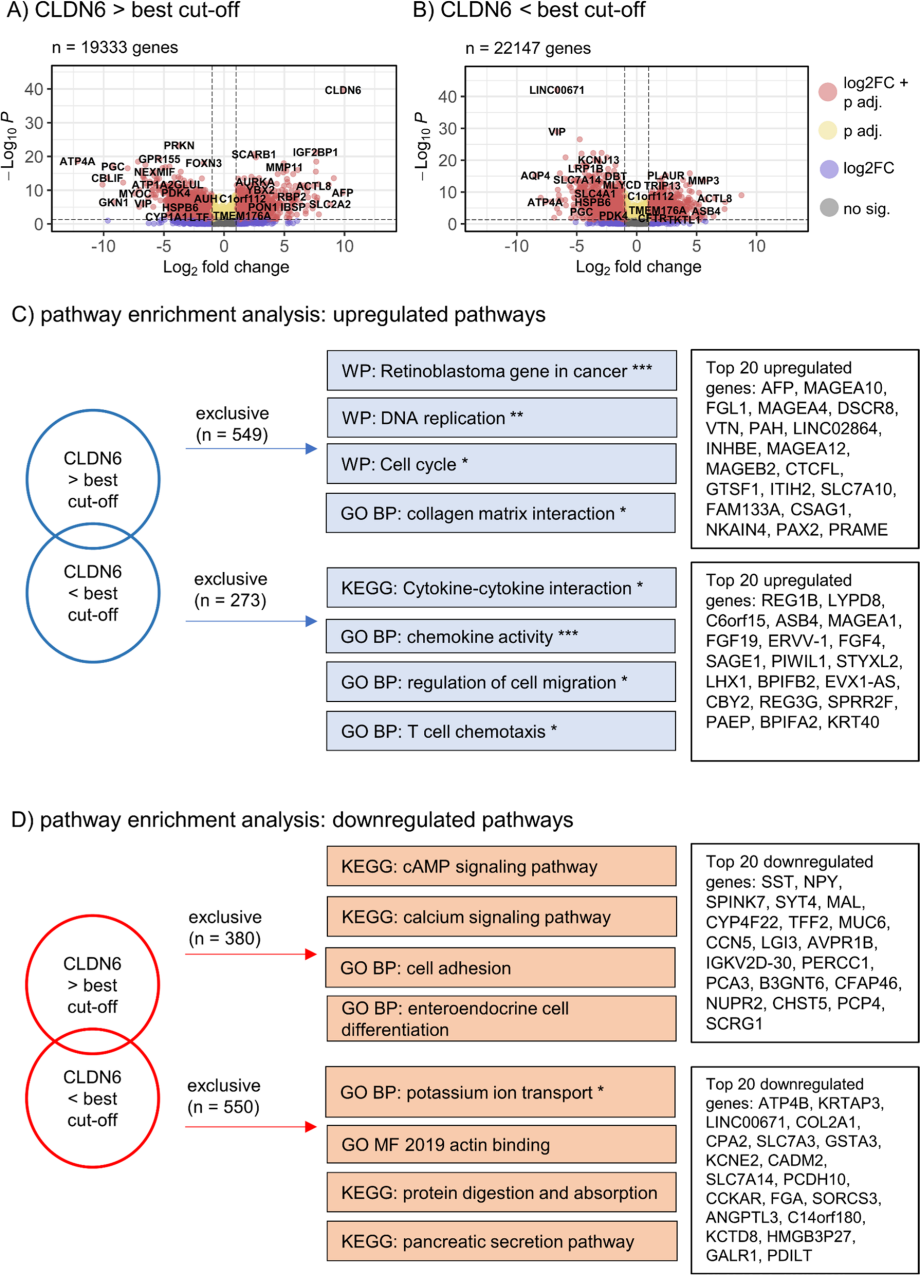
EACs with high or low CLDN6 expression have distinct biological profiles. **A**, **B** Volcano plots of dysregulated genes in EAC compared to benign tissue dichotomized by CLDN6 expression above and below the best survival-associated cutoff. A log2 fold change (log2FC) ≥ 1.5 was considered for upregulation, and a log2FC ≤ -1.5 for downregulation. An adjusted p-value < 0.05 (Wald test, Bonferroni correction) was considered significant. **C** Exclusively upregulated pathways in EAC with CLDN6 expression above and below the best survival-associated cutoff and the top 20 exclusively upregulated genes in both subgroups, respectively. **D** Exclusively downregulated pathways and top 20 downregulated genes in CLDN6-high and -low EAC. *CLDN6* Claudin 6, *WP* WikiPathway Human 2021, *GO BP* GO Biological Process 2021, *KEGG* KEGG 2021 Human, *GO MF* GO Molecular Function 2021.P. P-values and levels of significance: *** p adjusted < 0.001, ** p adj. < 0.01, *p adj. < 0.05 (Enrichr pathway allocation with Bonferroni correction)

DESeq differential gene expression analysis was performed for EAC and GAC subgroups with CLDN6 expression above and below the best cut-off (Table 4). Genes that were exclusively up- or downregulated in tumors with high or low CLDN6 expression were allocated to specific pathways using the Enrichr platform (Fig. 2).

**Table 4:** Dysregulated gene expression in EAC and GAC cohorts

	EAC		GAC	
Genes (%)	CLDN6 > best cutoff	CLDN6 < best cutoff	CLDN6 > best cutoff	CLDN6 < best cutoff
Dysregulated	19333 (100.0)	22147 (100.0)	19617 (100.0)	22508 (100.0)
Significant^a^	6255 (32.4)	7413 (33.5)	12227 (62.3)	13652 (60.7)
Upregulated^b^	1069 (5.5)	1239 (5.6)	1326 (6.8)	1215 (5.4)
Downregulated^b^	1069 (5.5)	793 (3.6)	1530 (7.8)	1521 (6.8)
Upregulated, exclusive^c^	549 (2.8)	273 (1.2)	577 (2.9)	466 (2.1)
Downregulated, exclusive^c^	380 (2.0)	550 (2.5)	409 (2.1)	400 (1.8)

*CLDN6* claudin 6, *EAC* esophageal adenocarcinoma, *GAC* gastric adenocarcinoma

^a^a p-value adjusted < 0.05 was interpreted as significant (Ward test, Bonferroni correction)

^b^a log2 Fold Change ≥ 1.5 was considered upregulated, a log2 Fold Change ≤—1.5 a downregulation

^c^genes exclusively up or downregulated in each tumor subgroup (CLDN6 > */* < best cutoff)

In EAC cases with high CLDN6 expression, 549 genes were exclusively upregulated. Among the top 20 upregulated genes were the alpha fetoprotein (*AFP*) gene*,* several genes of the melanoma antigen (MAGE) protein family, and other melanoma-associated genes (*MAGE-A4, -10, -12, -B-2; DSCR8*, *PRAME*). The Enrichr tool allocated these genes, among others, to pathways regulating the cell cycle and DNA replication (*CDT1, MCM4, MCM2, CDK2, CDK4, E2F1* and others, Fig. 2C). These tumors furthermore overexpressed several collagens, including *COL1A1, -9A1, -12A1* and *-27A1,* as well as metalloproteinases (*MMP13, MMP17*). *ERBB2* (Her2) was also upregulated in these tumors*,* as well as the gene *SALL4.* We observed exclusive downregulation of 380 genes regulating calcium signaling, ion channel activity, cell adhesion, and epithelial differentiation, although none of these pathway allocations were significant in the Enrichr analysis (Fig. 2D).

EAC tumors with CLDN6 expression below the best cut-off displayed a different expression profile. A total of 273 genes were exclusively upregulated that were significantly associated with cell migration and cytokine interaction pathways (*CEACAM1, IL1B, IL17F, CXCL9, CXCL11, CXCL16, CCL24, CCL26*)*.* EACs exclusively showed significant downregulation of 550 genes involved in secretory pathways, regulation of protein metabolism, actin binding, and potassium ion transport (*KCNJ11, KCNJ12, KCN13, KCNH7*).

**Fig. 3:**
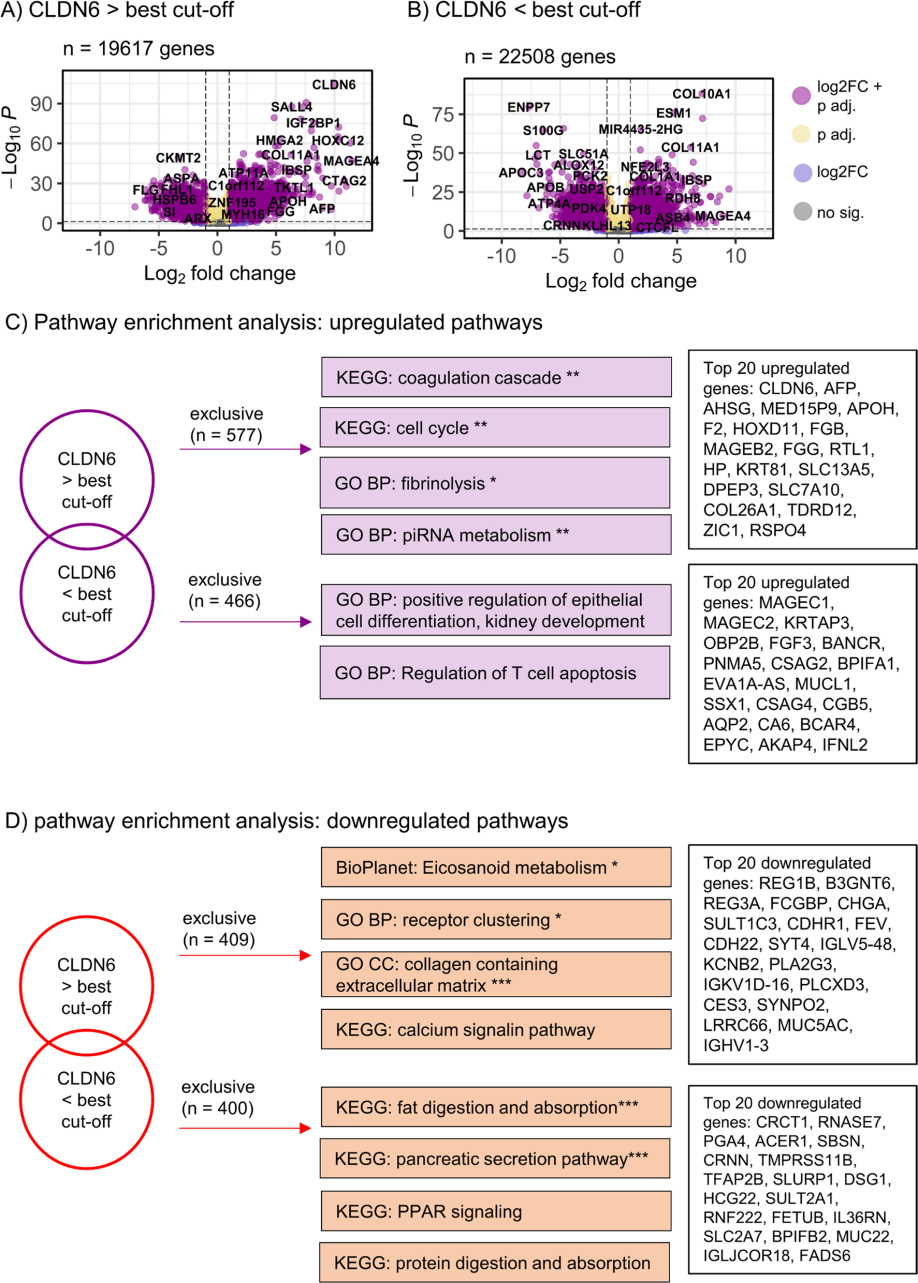
GACs with high or low CLDN6 levels express distinct biological pathways. **A**, **B** Volcano plots of dysregulated genes in GAC compared to benign tissue dichotomized by CLDN6 expression above and below the best survival-associated cutoff. A log2 fold change (log2FC) ≥ 1.5 was considered for upregulation, and a log2FC of ≤ -1.5 for downregulation. An adjusted p-value < 0.05 (Wald test, Bonferroni correction) was considered significant. **C**, **D** Exclusively upregulated and downregulated pathways in GAC with CLDN6 expression above and below the best survival-associated cutoff and the top 20 exclusively up- and downregulated genes in both subgroups. *CLDN6* Claudin 6, *GO BP* GO Biological Process 2021, *KEGG* KEGG 2021 Human, *GO CC* GO Cellular Compartment 2021, *BioPlanet* BioPlanet 2019; p-values and levels of significance: *** p adjusted < 0.001, ** p adj. < 0.01, *p adj. < 0.05 (Enrichr pathway allocation with Bonferroni correction)

As in EAC, GACs with high CLDN6 levels showed upregulation of *AFP* and *ERBB2* (Her2) genes (Fig. 3). Other genes regulating tight junctions (*CLDN1, CLDN19*) were upregulated. We additionally noted overexpression of genes regulating cell cycle (*E2F3, MCM7, CDC20, CDC25A* and others), fibrinolysis, and coagulation (*FGA, FGB, VTN, FGG*) (Fig. 3C). The 409 exclusively downregulated genes were allocated to collagen matrix interactions and receptor interactions (*MADCAM1, MUSK, PIGR*) (Fig. 3D). In GACs with lower CLDN6 levels, upregulation of genes involved in renal epithelial formation and T-cell apoptosis was observed, although this allocation was not significant in the Enrichr analysis. However, these tumors, as with the EAC specimens with lower CLDN6 expression, displayed downregulated secretion and protein metabolism pathways as well as decreased expression of genes regulating fat metabolism and genes of the peroxisome proliferator-activated receptor pathway.

### CLDN6 protein is expressed in a small subgroup of EAC and GAC specimens in the TMA cohorts

**Fig. 4:**
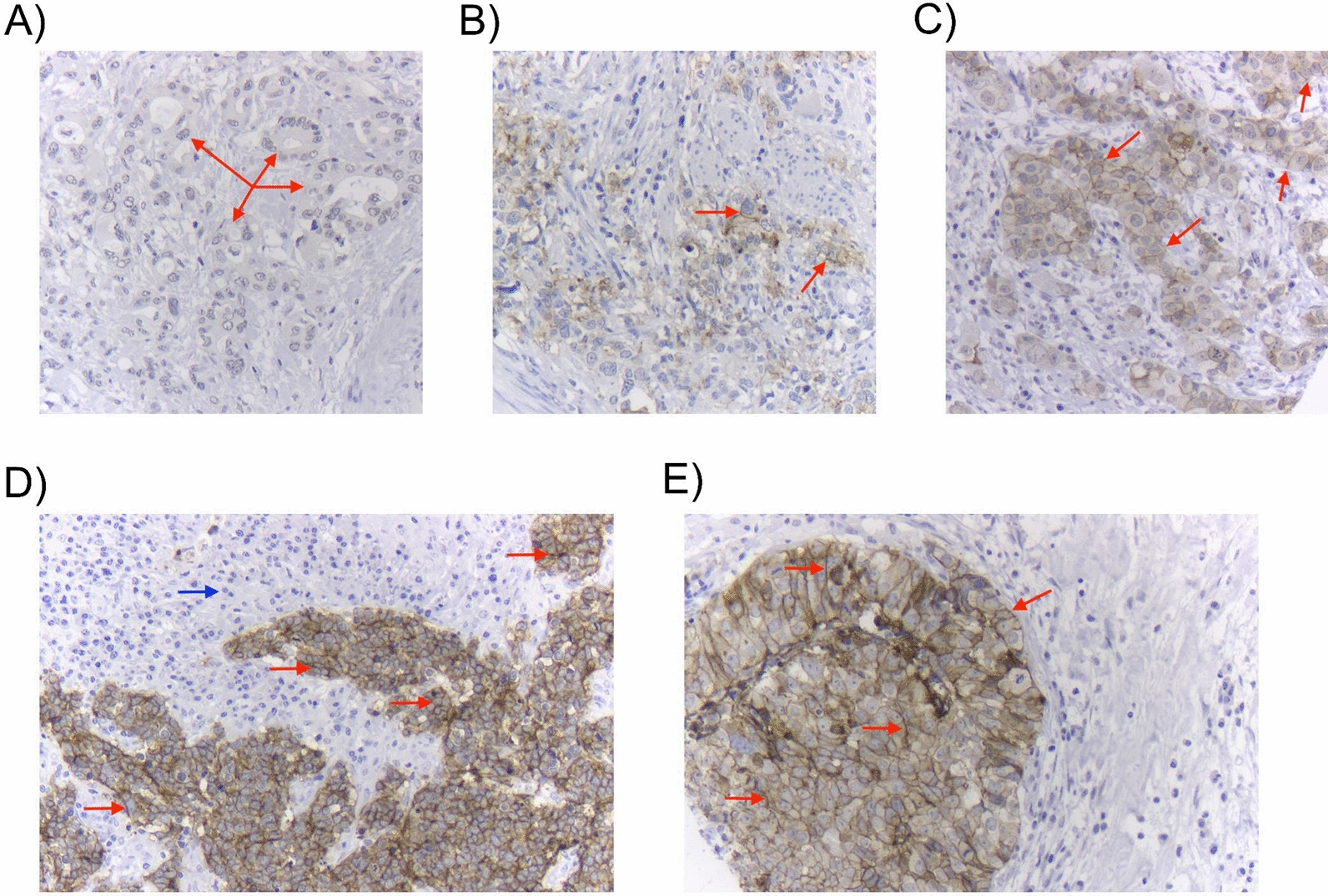
Adenocarcinomas of the upper gastrointestinal tract, immunohistochemically stained for CLDN6. Red arrows indicate tumor cells and highlight membrane staining. **A** CLDN6-negative adenocarcinoma. **B** Adenocarcinoma with only weak focal expression of CLDN6 (score 1 +). **C** Low-grade CLDN6-positive adenocarcinoma (focally 2 +) **D**, **E** Two different carcinomas expressing high levels of CLDN6 (focally 3 +); The blue arrow in **D** represents peritumoral, CLDN6-negative inflammatory cells; Magnification 200x. *CLDN6* claudin 6

Immunostaining for CLDN6 protein expression revealed that most of the EAC and GAC specimens from the tissue microarray cohorts were negative for CLDN6 with only 67 EAC samples (8.3%) and 18 GAC samples (3.0%) showing any CLDN6 expression (Fig. 4, Table 5).

**Table 5:** Staining for CLDN6 protein in EAC and GAC cohorts

	EAC (n = 808)	GAC (n = 589)
CLDN6 expression	n (%)	n (%)
0–Negative	736 (91.7)	571 (97.0)
1–Weak	48 (6.0)	12 (2.0)
2–Moderate	14 (1.7)	6 (1.0)
3–Strong	5 (0.6)	0 (0.0)
Positive (1–3)	67 (8.3)	18 (3.0)
1–9% of cells	4 (0.5)	9 (1.5)
10–19% of cells	16 (2.0)	3 (0.5)
≥ 20% of cells	47 (5.9)	6 (1.0)
≥ 50% of cells	24 (3.0)	1 (0.2)

*EAC* esophageal adenocarcinoma, *GAC* gastric adenocarcinoma

In the total EAC TMA cohort, CLDN6 expression was not associated with patients’ age and sex (data not shown). We did not observe any interdependencies between CLDN6 expression and tumor stage (pT), lymph node stage (pN), lymph vessel, blood vessel, and perineural invasion or the application of neoadjuvant treatment. No correlation was observed between Her2 status (ERBB) and the CLDN6 expression. In the primary EAC surgery cohort (no neoadjuvant treatment), CLDN6-positive tumors more often metastasized to the lymph nodes (statistical trend, p = 0.07); An association of CLDN6 expression with age, sex, other histopathological parameters, and American Joint Committee on Cancer (AJCC) grade was not observed (data not shown). This was also the case in the EAC cohort with neoadjuvant (radio) chemotherapy in total and subdivided by CROSS and FLOT regimen (data not shown).

In all GAC cohorts, we did not observe any correlations between CLDN6 expression and patients’ age, sex, and any histopathological parameters, neither in the total cohort nor the treatment-naïve or pre-treated subgroups. The CLDN6 expression and Her2 status (ERBB) were not associated (Fisher’s exact test, p = 0.64). In GAC cohorts, unlike for CLDN6 mRNA expression in the TCGA cohort, we did not observe a correlation between CLDN6 protein expression and molecular subtype (Fisher’s exact test, p = 0.29).

### CLDN6 protein expression is associated with lower OS in EAC and GAC TMA cohorts

**Fig. 5:**
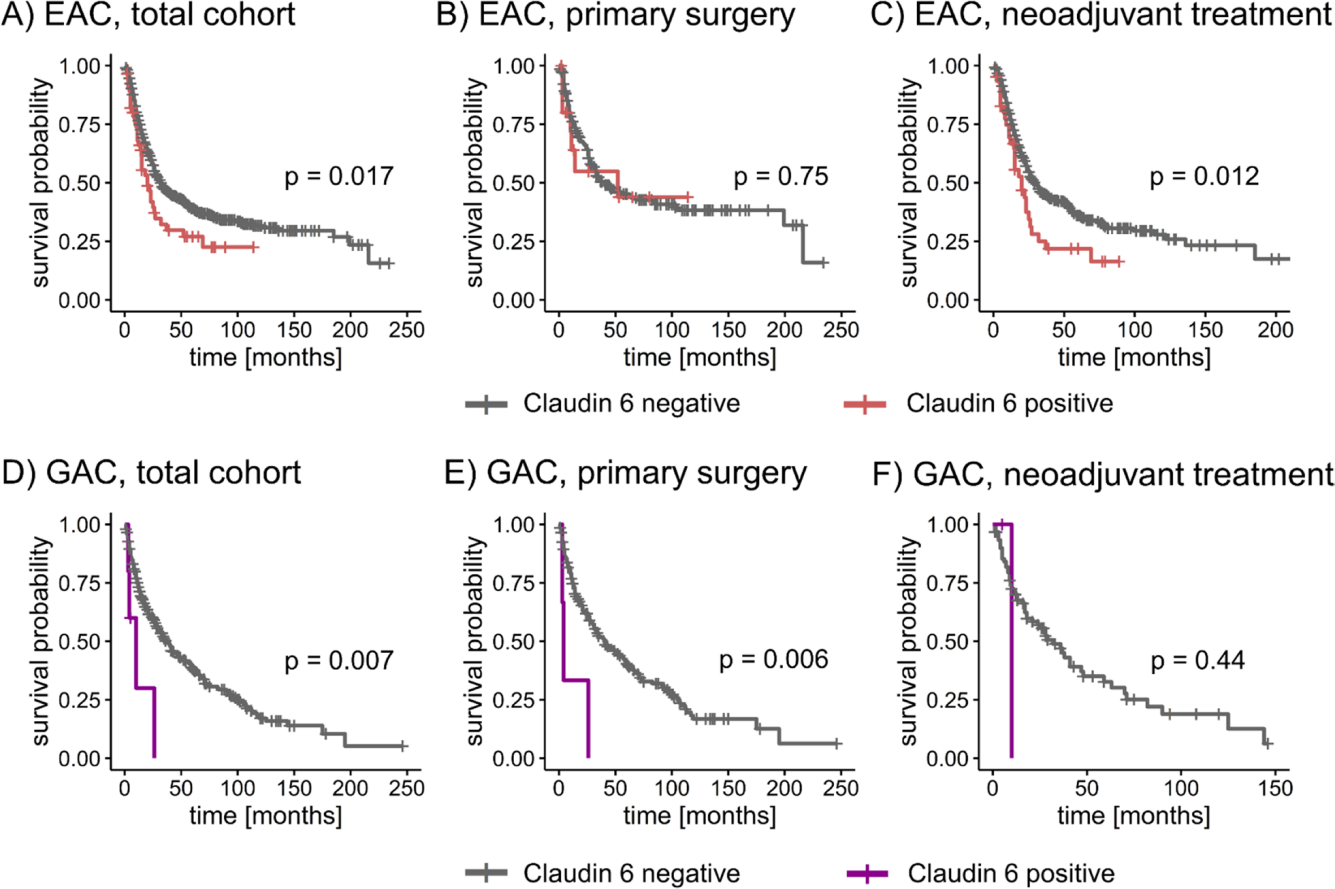
CLDN6 expression is associated with shorter OS in EAC and GAC cohorts. **A**-**C** OS in patient cohorts of EAC dichotomized by presence of CLDN6-negative and CLDN6-positive tumors. Primary surgery cohort = patients without neoadjuvant treatment before surgery; Neoadjuvant treatment cohort = patients treated with CROSS and FLOT regime. **D**–**F** OS in patient cohorts of GAC dichotomized by CLDN6 expression (positive vs. negative); Neoadjuvant treatment cohort included patients treated with PFL, MAGIC and FLOT regimens; A p-value < 0.05 (log-rank test) was considered significant. *CLDN6* claudin 6, *CROSS* Chemo-radiotherapy for Oesophageal cancer followed by Surgery Study, *EAC* esophageal adenocarcinoma, *FLOT* fluorouracil-leucovorin-oxaliplatin-docetaxel, *GAC* gastric adenocarcinoma, *MAGIC* Medical Research Council Adjuvant Gastric Infusional Chemotherapy, *OS* overall survival, *PFL* Cisplatin, 5-Fluorouracil and Leucovorin

In the total EAC cohort, we observed a significantly shorter OS for patients with CLDN6-positive tumors compared with CLDN6-negative tumors (median OS: 20.0 months vs. 33 months, log-rank test p = 0.017; censored at the last time point of follow-up) (Fig. 5A). This was also demonstrated in patients who received neoadjuvant treatment (median OS: 20 months vs. 30 months, log-rank test p = 0.012, Fig. 5C), but not in the primary surgery cohort (p = 0.75, Fig. 5B).

In the GAC cohort, patients with CLDN6-positive tumors had significantly shorter OS when compared with patients with CLDN6-negative tumors (median OS: 10 months vs. 37 months, log-rank test p = 0.007) (Fig. 5D). The same reduced OS was displayed in the patient cohort with primary surgery (median OS: 4 months vs. 40 months, log-rank p = 0.006) (Fig. 5E). In the cohort with neoadjuvant treatment, only four tumors expressed CLDN6, and a statistical difference in OS was not demonstrated (Fig. 5F).

### CLDN6 protein expression is an adverse prognostic factor in patients with EAC and GAC

CLDN6 expression was associated with a adverse outcome in the total EAC cohort (HR: 1.52, 95% CI 1.08–2.14, p = 0.018). In the multivariate analysis, CLDN6 expression remained associated with worse prognosis when other significant covariates were included (p = 0.01, Table 6). In the EAC subgroup treated with neoadjuvant therapy, CLDN6 positivity was also an adverse prognostic factor in the univariate analysis (HR: 1.63, 95% CI 1.11–2.4, p = 0.013). CLDN6 remained significantly correlated with worse outcomes in this subgroup in the multivariate analysis (p = 0.037, Table 6).

**Table 6:** Multivariate analysis for CLDN6 expression in EAC and GAC

	**EAC**		**GAC**	
Covariate	HR (95% CI)	p-value	HR (95% CI)	p-value
Total cohort				
Age	1.02 (1.01–1.03)	**0.002**	1.01 (0.99–1.02)	0.28
pT	1.32 (1.10–1.59)	**0.004**	1.64 (1.30–2.07)	** < 0.001**
pN	2.28 (1.70–3.06)	** < 0.001**	0.92 (0.61–1.37)	0.66
L	1.31 (1.00–1.70)	**0.046**	1.92 (1.25–2.94)	**0.003**
V	0.95 (0.64–1.41)	0.81	1.17 (0.74–1.88)	0.50
Pn	1.23 (0.91–1.66)	0.17	0.90 (0.60–1.35)	0.61
M	2.0 (1.21–3.30)	**0.007**	1.89 (1.23–2.92)	**0.004**
Her2 status (ERBB)	0.68 (0.42–1.11)	0.12	1.41 (0.88–2.26)	0.15
Neoadjuvant treatment	1.21 (0.92–1.59)	0.17	1.13 (0.79–1.62)	0.50
CLDN6 expression	1.75 (1.14–2.68)	**0.01**	2.74 (0.98–7.69)	**0.028**
Primary surgery cohort				
Age	1.03 (1.01–1.05)	**0.01**	1.00 (0.98–1.02)	0.92
pT	1.52 (1.07–1.91)	**0.01**	1.85 (1.21–2.11)	** < 0.001**
pN	2.28 (1.28–1.95)	**0.01**	0.86 (1.03–1.73)	0.55
L	1.85 (1.02–2.60)	**0.02**	2.23 (0.98–2.63)	**0.003**
V	0.93 (0.56–1.62)	0.82	1.06 (0.57–1.75)	0.85
Pn	0.97 (0.55–1.46)	0.91	0.99 (0.72–1.84)	0.97
M	2.16 (1.34–4.90)	**0.045**	1.86 (0.96–3.18)	**0.04**
AJCC Grade	1.27 (0.80–1.82)	0.31	0.60 (0.41–0.89)	**0.01**
Her2 status (ERBB)	0.92 (0.79–2.02)	0.82	1.33 (0.75 – 2.35)	0.33
CLDN6 expression	1.12 (0.4–3.18)	0.82	4.21 (1.26–14.07)	**0.02**
Neoadjuvant cohort				
Age	1.01 (0.99–1.02)	**0.42**	1.02 (0.99–1.05)	0.22
pT	1.16 (0.90–1.50)	0.24	1.54 (0.98–2.41)	0.06
pN	1.48 (1.26–1.72)	** < 0.001**	1.02 (0.46–2.27)	0.96
L	1.06 (0.75–1.50)	0.74	2.17 (0.96–4.89)	0.06
V	0.86 (0.49–1.51)	0.60	1.26 (0.51–3.07)	0.62
Pn	1.14 (0.77–1.68)	0.5	0.69 (0.30–1.56)	0.37
M	2.9 (1.02–8.25)	**0.046**	2.45 (1.17–5.15)	**0.02**
Her2 status (ERBB)	0.53 (0.27–1.04)	0.06	1.31 (0.48–3.58)	0.59
CLDN6 expression	1.73 (1.03–2.91)	**0.037**	2.80 (0.32–24.34)	0.35

The bold values represent significant p-values (< 0.05)

*AJCC* American Joint Committee on Cancer, *EAC* esophageal adenocarcinoma, *CI* confidence interval, *GAC* gastric adenocarcinoma, *L* lymph vessel invasion, *HR* hazard ratio, *M* distant metastasis, *NA* Not analyzed, *pT* tumor stage, *pN* lymph node stage, *Pn* Perineural invasion, *V* blood vessel invasion

In the total GAC cohort, CLDN6 expression was also associated with a worse outcome in the univariate analysis (HR: 3.76, 95% CI 1.38–10.22, p = 0.01) and multivariate analysis (p = 0.028, Table 6). In contrast to the EAC cohorts, CLDN6 expression was associated with a worse outcome in univariate analysis in treatment-naïve patients (HR: 4.633; 95% CI 1.46–14.73; p = 0.009), while it was not a significant prognostic factor in the pre-treated subgroup (p = 0.44). In multivariate analysis, like in the total GAC cohort, CLDN6 expression was significantly associated with an adverse outcome in the cohort of treatment-naïve patients (p = 0.02, Table 6).

## Discussion

CLDN6 has previously been identified as a potential prognostic marker for GAC. Kohmoto et al. demonstrated in the TCGA GAC cohort, which was also used in this study, that high expression of CLDN6 mRNA is an adverse prognostic factor and is associated with the CIN subtype [18]. We confirm these findings and furthermore used a dichotomization approach with a survival-associated cutoff to analyze changes in gene expression in tumors with high or low CLDN6 expression. Pathway analysis was subsequently conducted for genes that were up or downregulated in EACs and GACs. For GACs, pathway enrichment analyses were reported recently by Dwivedi et al. who used a slightly different dichotomization approach for their analysis, but described very similar observations [30]. In their study, CLDN6-enriched GACs expressed high levels of cancer/germline antigens (MAGE-A, -B), which were originally detected to be dysregulated in melanoma cells and have been linked to TP53 degradation, aggressive tumor growth, and a poorer prognosis [31]. In the current study, we also observed elevated expression of melanoma antigens (MAGE-A4, -10, -12, -B-2) in EACs. Since EACs have an extensive genetic overlap with the CIN subtype of GAC [32], to which most of the tumors with high CLDN6 expression belonged in our study, this finding strongly correlates to the Dwivedi results and also implicates that CLDN6-enriched subgroups of EAC and GAC will have a similar poor prognosis due to their similar genetic profile.

Other tight junction-associated genes (*CLDN1, -19*) and several metalloproteinases (*MMP13, -17*) were also upregulated in EACs with high CLDN6 expression. Torres et al. demonstrated that CLDN6 upregulation is linked to a consecutive upregulation of CLDN1, which co-localized with metalloproteinases (MMP2, -14) and was associated with a higher invasiveness and enhanced cell migration of EAC cell lines [33]. We furthermore observed exclusive upregulation of several collagens (COL1A1, -9A1, -12A1, -27A1) in esophageal cancers with high CLDN6 levels. Li et al. linked elevated collagen expression (COL1A2) to enhanced invasive growth and metastasis in two cell lines derived from squamous cell carcinoma [34]. Overexpression of COL1A1 in the tumor microenvironment has been linked to metastasis in colorectal and ovarian carcinoma as well as hepatocellular carcinoma [35–37]. Xiang et al. demonstrated that a knockdown of COL12A1 expression in GAC cell lines decreased cell migration, thus hindering the process of invasion and metastasis [38]. Although it was not in the top 20 of significantly upregulated genes, we also noted an increased expression of SALL4 in CLDN6-expressing EAC and GAC: SALL4 is associated with crucial biological functions in stem cells and was associated with tumorigenesis and epithelial-mesenchymal transition in esophageal squamous cell carcinoma and overall “stemness” in various other tumors [39, 40]. In summary, we not only confirmed the most recent findings for CLDN6-enhanced GAC, but could also describe distinct biological and genetic characteristics in EACs that also show a plausible overlap with CLDN6-enriched GACs. Some of the distinct upregulated pathways might also explain the decreased OS for patients with high CLDN6 expression.

Since CLDN6 is expressed in various solid tumors, but is essentially absent from healthy adult tissue, it has been highlighted as a potential therapeutic target [41]. We therefore further explored CLDN6 protein expression in EACs and GACs in TMA cohorts. While the expression of CLDN6 and its potential role and impact have been investigated before in smaller cohorts of GAC, which were mostly composed of Asian patients [18, 19, 42], the data on its expression in EAC are limited to absent. We therefore assembled the largest patient cohort of EAC and GAC cases of mostly Caucasian patients in a German treatment setting comprising more than 800 EACs and almost 600 GACs. We demonstrated that CLDN6 expression was not correlated with any histopathological parameter despite being associated with a poor prognosis. Kohmoto et al. described an interdependence between CLDN6 expression and enhanced tumor stage (pT) and lymph node metastasis in a smaller cohort of GAC; however, they could not confirm an independent prognostic impact in their multivariate analysis [18]. We demonstrated that CLDN6 expression is in fact not only associated with worse outcome in all GAC patients, but also in the treatment-naïve subgroup that received primary surgery. We furthermore demonstrated for the first time that a subset (8.3%) of EACs also expresses CLDN6 protein. This expression of CLDN6, while not associated with clinical and histopathological parameters, was correlated with a worse outcome, as observed in GAC. This was notable not only in the total cohort, but also in the cohort which received neoadjuvant treatment. In the treatment-naïve EAC cohort, this was not observed; One explanation might be the low number of CLDN6 expressing tumors (n = 22). Another possible explanation might be the high prevalence of tumors limited to the mucosa (pT1). Additionally, patients who receive primary surgery are, if the tumor is not mucosa-limited, usually not suitable anymore for highly aggressive (radio) chemotherapy due to age or comorbidities and might therefore have a worse outcome from the beginning, which might obscure the relevance of CLDN6 expression in this subgroup.

One of the limitations of this study is that it is a retrospective analysis and is based on a patient cohort from a single treatment center. Additionally, the analyses of small-scale tissue microarrays can obscure the heterogeneity of CLDN6 expression in large tumor samples. However, the large number of samples included in the current study addresses the problem of heterogeneity to a certain degree.

Several ongoing studies are currently investigating the therapeutic potential of the CLDN6 in several solid tumors. Reinhard et al. recently developed a CLDN6-directed chimeric antigen receptor (CAR) T-cell therapy in combination with a CLDN6-encoding mRNA vaccine, which led to an activation and stimulation of T cells specifically targeting CLDN6-expressing solid tumors [41]. While the final data are not yet available, first encouraging responses were observable in the treated patients with an overall response rate of 42% and a disease control rate of 92%, respectively [43]. Targeted therapies against other members of the claudin family are also being validated. Zolbetuximab, a chimeric antibody against claudin 18.2, showed prolonged OS and progression free survival (PFS) in highly CLDN18.2-expressing GEC/EAC patients when combined with epirubicin, oxaliplatin, and cabecitabine first line treatment in a Phase 2 study [44] and clinical benefit of adding zolbetuximab to CAPOX as well as mFOLFOX has been recently reported from two Phase 3 trials [45].

While in the current study, we showed that the mere presence of CLDN6 is an adverse prognostic factor, the response of tumors with weak and moderate CLDN6 expression levels to therapy has not been assessed so far. A Phase I/IIa study is currently ongoing that is investigating treatment with RNA vaccine-augmented CAR T cells against CLDN6 in various solid and advanced tumors (NCT04503278). This study may give us the first insights into the feasibility and the potential of CLDN6 as a target for new therapeutical approaches.

## Conclusions

Expression of CLDN6 is an adverse prognostic factor in EAC as well as GAC. Due to its exclusive expression in several solid tumors, CLDN6 may be a promising target, for example, for CAR T-cell therapy and other patient- and tumor-individualized approaches in these cancers of high medical need.

## Data Availability

The RNA-Seq data are openly available at TCGA data repository as well as the Broad Institute Firehose GDAC data base. The clinical data for the immunohistochemical analyses are available upon reasonable request.The CLDN6 antibody was validated by BioNTech corporation regarding several parameters and is at the moment used for patient selection for two separate clinical studies. Data regarding sensitivity/specificity are available upon reasonable request.

## Abbreviations

AJCC: American Joint Committee on Cancer
CIN: Chromosome-instable (subgroup of gastric adenocarcinomas)
CLDN6: Claudin 6
CROSS: Chemo-radiotherapy for Oesophageal Cancer followed by Surgery Study
EAC: Esophageal adenocarcinoma
(log)FC: Log Fold Change
FLOT: Fluorouracil, leucovorin, oxaliplatin and docetaxel (chemotherapy regime)
GAC: Gastric adenocarcinoma
IHC: Immunohistochemistry
MAGIC: Medical Research Council Adjuvant Gastric Infusional Chemotherapy (Epirubicin, Cisplatin, Fluorouracil)
MSI: Microsatellite-instable
PFL: Cisplatin, 5-fluorouracil and leucovorin (chemotherapy regime)
RNA-Seq: RNA sequencing
RSEM: RNA-Seq by expectation–maximization (normalization method)
TCGA: The Cancer Genome Atlas
TPM: Transcripts per million
